# Determinants of motivation to quit in smokers screened for the early detection of lung cancer: a qualitative study

**DOI:** 10.1186/s12889-018-6211-1

**Published:** 2018-11-20

**Authors:** Ben Young, Kavita Vedhara, Denise Kendrick, Roberta Littleford, John F. R. Robertson, Frank M. Sullivan, Stuart Schembri, Roshan das Nair, in collaboration with the ECLS study team

**Affiliations:** 1 Division of Primary Care, University of Nottingham, Nottingham, UK; 2 Tayside Clinical Trials Unit, University of Dundee, Dundee, UK; 3 Division of Medical Sciences and Graduate Entry Medicine, University of Nottingham, Derby, UK; 4 School of Medicine, University of St Andrews, St Andrews, UK; 5 Ninewells Hospital, Dundee, UK; 6 Division of Psychiatry & Applied Psychology, University of Nottingham, Nottingham, UK

**Keywords:** Smoking cessation, Early cancer detection, Lung cancer, Teachable moment, Thematic analysis

## Abstract

**Background:**

The promotion of smoking cessation within lung cancer screening could lead to benefits for smoking-related disease and improve cost-effectiveness of screening. Little is known about how smokers respond to lung cancer screening and how this impacts smoking behaviour. We aimed to understand how lung cancer screening influences individual motivations about smoking, including in those who have stopped smoking since screening.

**Methods:**

Thirty one long-term smokers aged 51–74 took part in semi-structured interviews about smoking. They had been screened with the EarlyCDT-Lung Test (13 positive result; 18 negative) as part of the Early Cancer Detection Test Lung Cancer Scotland Study. They were purposively sampled for interview based on their self-reported post-screening smoking behaviour. Eleven participants had stopped smoking since screening. Verbatim interview transcripts were analysed using thematic analysis.

**Results:**

Two key overarching themes were *interpretations of screening test results* and *emotional responses* to those interpretations. Participants’ understanding of the risk implied by their test result was often inaccurate, for example a negative result interpreted as an ‘all-clear’ from lung cancer and a positive result as meaning lung cancer would definitely develop. Those interpretations led to e*motional responses* (fear, shock, worry, relief, indifference) influencing motivations about smoking. Other themes included *a wake-up call* causing changes in perceived risk of smoking-related disease, a feeling that *now is the time to stop smoking* and *family influences*. There was no clear pattern in smoking motivations in those who received positive or negative test results. Of those who had stopped smoking, some cited screening experiences as the sole motivation, some cited screening along with other coinciding factors, and others cited non-screening reasons. Cues to change were experienced at different stages of the screening process. Some participants indicated they underwent screening to try and stop smoking, while others expressed little or no desire to stop.

**Conclusions:**

We observed complex and individualised motivations about smoking following lung cancer screening. To be most effective, smoking cessation support in this context should explore understanding of screening test results and may need to be highly tailored to individual emotional responses to screening.

**Electronic supplementary material:**

The online version of this article (10.1186/s12889-018-6211-1) contains supplementary material, which is available to authorized users.

## Background

Tobacco use is responsible for more than five million deaths a year worldwide [1] and awareness of the link between smoking and lung cancer is high [2]. Lung cancer screening using low-dose computed tomography (CT) is recommended in the USA for those aged 55–80 with a 30 pack year smoking history [3]. Smokers who engage in lung cancer screening may be more motivated to quit [4] and screening could further influence smoking thoughts, motivations and behaviour via a ‘teachable moment’ or a ‘license to smoke’.

Predictors of attempts to stop smoking include having made a past quit attempt, lower cigarette dependence, higher motivation and intention to quit and belief in the harm caused by smoking [5]. Qualitative research has further described how health concerns can lead to quit attempts, often combined with other internal or environmental influences [6]. This work was conducted outside of the context of lung cancer screening.

Of three randomised lung cancer screening studies that have compared smoking in screened and control groups, one observed higher quit rates in the screened group at 2 weeks and at 2 years [7] and two found no long-term effect of lung cancer screening on smoking [8, 9]. Groups screened with CT or chest X-ray in the National Lung Screening Trial reported similar smoking cessation rates after 3 years [10]. Screened participants who receive an abnormal screening result appear more likely to quit and less likely to relapse [7, 10–14]. Increasing rates of smoking cessation in screening patients could lead to benefits for smoking-related disease and cost-effectiveness of screening [15]. Professional and medical guidelines recommend the integration of smoking cessation interventions into lung cancer screening programmes [16, 17] although pilot studies of this approach provide mixed findings of effectiveness [18–22].

There is a lack of evidence on how support should be provided to most effectively promote smoking abstinence in those screened for lung cancer [23, 24]. In particular, little is known about how lung cancer screening influences individual motivations about smoking. Two studies have used qualitative methods to explore smoking in this context. The first study found that nobody within a sample of 35 National Lung Screening Trial participants had stopped smoking more than a year after screening but some had reduced their smoking. Structured interviews were used and factors influencing motivations about smoking were not explored in depth [25]. The second study reported three of 37 participants had quit smoking since screening, one of whom said the offer of screening had changed their thoughts about smoking and another said the finding of nodules motivated them to quit [26]. Others reported a lack of urgency to quit, sometimes citing the monitoring of CT findings as a reason. To add to this evidence that lung cancer screening can both increase and decrease motivation to quit, a qualitative investigation is needed of factors influencing the motivations of those that decide to attempt to stop smoking after screening and those that decide not to. There has been no in-depth study to date of individuals who have stopped smoking after being screened for lung cancer.

Based on previous literature on this topic we expected that lung cancer screening might involve experiences that in some way promote or inhibit attempts to stop smoking. The aim of our study is to explore motivations about smoking in smokers screened for the early detection of lung cancer, including those who stop smoking after screening, to better understand how screening impacts motivations to stop or continue smoking and how cessation support can promote smoking abstinence in this context.

## Methods

### Study design

We conducted a qualitative study as part of the Early Cancer Detection Test - Lung Cancer Scotland (ECLS) study, a randomised controlled trial evaluating the effectiveness of a blood test (EarlyCDT-Lung) to detect lung cancer early [27]. ECLS study participants lived predominantly in the most deprived areas of the three study regions of Greater Glasgow and Clyde, Tayside and Lanarkshire, in Scotland, UK. Participants were at increased risk of lung cancer due to having at least a 20 pack-year smoking history or a family history of lung cancer combined with a smoking history representing an equivalent risk. Blood samples were taken from all participants and those randomised to the screening arm were screened for levels of autoantibodies to lung cancer, which enabled risk stratification for the targeting of chest CT scans. Study materials informed participants that EarlyCDT-Lung detects 40 of every 100 cases of lung cancer and that eight out of every nine people receiving a positive test result do not have lung cancer. Those with a negative test result were notified by a letter stating that between 98 and 99 out of every 100 people with a negative test do not have lung cancer at the time of the test, and inviting them to contact the research centre if they have any questions. Those with a positive test result discussed the implications with a research nurse face-to-face or by telephone. They were informed the CT scan might detect pulmonary nodules and that in the majority of people they are of no health concern. They underwent a chest X-ray and CT scan and, if lung cancer was not diagnosed, they received four further CT scans at 6 month intervals. Smokers did not routinely receive cessation support as part of the ECLS study in order to prevent stigmatisation as a smoker indicated as a potential recruitment barrier by pre-trial focus groups [28]. However, they could be asked by the research nurse at their initial visit if they would like information on smoking cessation or referral on to an appropriate service. Participants were reminded of the importance of visiting their general practitioner if they experience named lung cancer symptoms.

### Recruitment and data collection

**Fig. 1:**
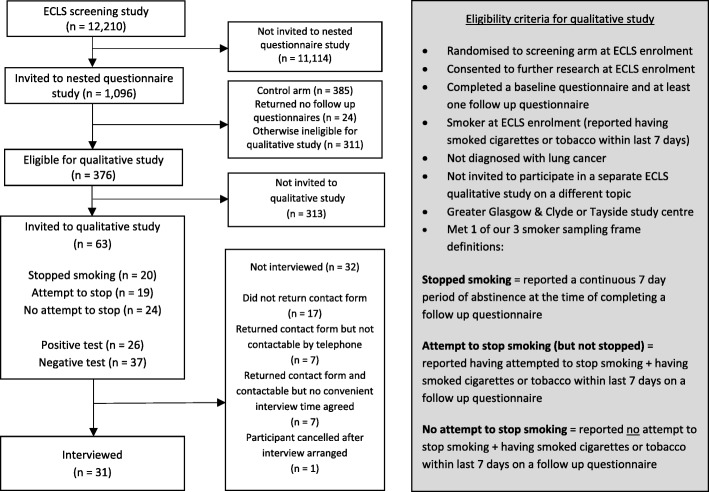
Participant flowchart with eligibility criteria and smoker sampling frame definitions

We sampled participants for the qualitative study from a subset of 1043 ECLS study participants taking part in a nested questionnaire study exploring psychological and behavioural responses to screening. Questionnaires collected self-reported data on current smoking status and recent attempts to stop smoking. A quota sampling approach was adopted with the aim of recruiting ten people who had stopped smoking since screening, ten who had attempted to stop but were still smoking, and ten who had not attempted to stop. Our definitions for each category of this sampling frame and other eligibility criteria are shown in Fig. 1. We aimed to recruit participants from across two ECLS study regions (Lanarkshire had not yet begun recruiting) and participants who received positive and negative EarlyCDT-Lung results. This approach was to ensure a diverse range of screening experiences and behavioural responses were represented in our sample. Within each quota we took a convenience sampling approach: eligible individuals who had most recently returned a follow up questionnaire were sampled in advance of scheduled research visits. They were sent an invitation letter, information leaflet and a contact form to return in a prepaid envelope to express interest in taking part. The leaflet explained that we were investigating what people think about smoking after lung cancer screening and emphasised that the purpose of the study was not to try to stop them smoking. This aimed to avoid discouraging those who did not want to stop smoking from taking part. On return of a contact form a researcher telephoned the participant to explain the study, answer any questions and arrange a convenient time for an interview. Participants completed a consent form before the interview. They were advised that the researcher held no strong feelings about smoking and was simply interested in their thoughts and feelings. Semi-structured, face-to-face interviews began with questions about smoking histories and general ECLS study experiences, then focused on motivations and decisions made about smoking since ECLS study enrolment and explored reasons for those. The interview guide is available in Additional file 1. Interviews also covered topics not reported here: barriers and facilitators to smoking abstinence and attitudes and preferences for smoking cessation support within lung cancer screening. Interviews were audio recorded and transcribed (anonymised) verbatim. All participants were offered a £5 multi-store gift voucher to thank them for participation. They were already receiving a series of identical £5 vouchers for completing the questionnaires so the interview incentive formed part of a larger available remuneration package.

### Analysis

Transcripts were analysed in NVivo software using a process of inductive and deductive thematic analysis [29, 30]. This involved familiarisation with the data, systematic coding of data, generation of a set of initial codes, sorting of codes into structures containing overarching themes and their subthemes (using separate a priori structures to address distinct aims of the research), reviewing and refining themes and finally, defining and further refining themes to create a coherent and internally consistent account of the data. One coder analysed all the data and a second senior investigator checked samples of transcripts in discussion with the coder at various points throughout the analysis. This enabled the revision of codes and their structures and the development of themes. We continued the coding process until all data had been systematically coded. During this process any new concepts identified prompted the review of all interview transcripts to check for the presence of that concept. We followed the criteria of Seale et al. [31] to ensure quality of the analysis and write up. We report here the subset of data relating to motivations about smoking.

## Results

Of 12,210 ECLS study participants, 63 were sent an invitation to the qualitative study, 46 (73%) responded and 31 (49%) were interviewed (Fig. 1). Based on participant preference, 27 interviews took place in participants’ homes and four in a private room in a clinical research facility. Average interview length was 40 min (range 16–80). Characteristics of participants and of the source population are shown in Table 1. Interviewees were comparable to the source population on most characteristics. They were, however, less likely to live in the most deprived areas and were more likely to have been intending to stop smoking at ECLS study enrolment. The timing of interviews in relation to ECLS study events are shown in Table 2. Most interviews took place within 5 months of screening and, for those with a positive result, most took place before their 6 month ECLS study schedule CT scan. During their interviews 11 participants reported having stopped smoking since lung cancer screening. The quotes presented were selected as being the most succinct and representative data extract(s) of each theme.

**Table 1:** Participant and source population characteristics at ECLS study enrolment

	Interviewed (*n* = 31)		Source population (*n* = 376) (screened ECLS study participants completing follow-up questionnaires who smoked at enrolment)	
	n (%) [missing]	Median Range [missing]	n (%) [missing]	Median Range [missing]
Age (years)		58 51–74 [0]		59 50–75 [0]
Gender				
Man	15 (48.4)		198 (52.7)	
Woman	16 (51.6) [0]		178 (47.3) [0]	
UK region				
Greater Glasgow & Clyde	21 (67.7)		268 (71.3)	
Tayside	10 (32.3) [0]		108 (28.7) [0]	
Ethnicity				
White Scottish or White British	30 (100) [1]		366 (98.4) [4]	
Scottish Index of Multiple Deprivation				
1 (most deprived quintile)	10 (32.3)		164 (43.7)	
2	8 (25.8)		86 (22.9)	
3	6 (19.4)		50 (13.3)	
4	5 (16.1)		44 (11.7)	
5 (least deprived quintile)	2 (6.5) [0]		31 (8.3) [1]	
At least one parent or sibling with a lung cancer diagnosis				
Yes	6 (19.6)		90 (23.9)	
No	25 (80.7) [0]		286 (76.1) [0]	
Smoking pack year history		40 20–175 [0]		37 2–175 [0]
Average no. cigarettes smoked a day		15 2–60 [0]		17 0–136 [2]
Attempted to stop smoking in last 6 months				
Yes	9 (31.0)		95 (25.8)	
No	20 (69.0) [2]		273 (74.2) [8]	
Intend to stop smoking in next 4 weeks				
Yes	10 (32.3)		90 (24.2)	
Don’t know	10 (32.3)		100 (26.9)	
No	11 (35.5) [0]		182 (48.9) [4]	
EarlyCDT-Lung result				
Positive	13 (41.9)		164 (43.6)^a^	
Negative	18 (58.1) [0]		212 (56.4) [0]	

^a^EarlyCDT-Lung results in source population not representative of all ECLS study participants due to higher sampling rate of positive test vs. negative test participants for questionnaire study

**Table 2:** Interview timings in the context of the ECLS study

	Median Range
Days since EarlyCDT-Lung screening	146 110–254
Days since EarlyCDT-Lung result letter sent	126 79–228
Positive test participants (*n* = 13):	
Days since ECLS first CT scan	123 72–209
Days before ECLS schedule CT scan (*n* = 11 [85%])	58 12–116
Days after ECLS schedule CT scan (*n* = 2 [15%])	n/a 14; 28

The two key overarching themes extracted in relation to motivations about smoking were *interpretations of screening test results* and *emotional responses* to those interpretations.

### Interpretations of screening test results

Participants’ interpretations of screening test results were a perceptual filter through which screening influenced motivations about smoking. Understanding of the risk implied by test results was often inaccurate; for example, a negative result interpreted as being ‘all clear’:

P20^1^: *And you say ‘well I’ve got a chance, I've not got it so this would be a good time to stop, I’ve just been given the all-clear’.* (Man,^2^ 56,^3^ negative,^4^ not stopped smoking^5^)

Here a positive result is interpreted as meaning lung cancer would definitely develop in the future:

P27: *I thought when it was positive that it was there and it was ‘you’ll get lung cancer,’ I thought that’s the way it worked.* (Woman, 63, positive, stopped smoking)

Some interpretations demonstrated a more accurate understanding:

P7: *The positive markers were coming up in my blood and look I read everything and it explained about it could be a false positive.* (Woman, 53, positive, stopped smoking)

P6: *It’s a one in nine chance over the next 2 years ... I thought well one in nine, that’s roughly the same risk of one in eight smokers getting lung cancer anyway, it’s just a pretty short timescale they’ve given me but it’s pretty good odds.* (Woman, 71, positive, stopped smoking)

Other interpretations involved confusion about the presence or absence of lung cancer:

P12: *I mean to be honest I couldnae [could not] sit and tell you right now whether I’ve got cancer or whether I’m getting it. I know I tested positive for it, so what does that mean? Have I got it, or am I going to get it? ... But through my own fault it’s confusing, cause I don’t want to know. So you just get up every day and continue to smoke cause you think to yourself, ‘well I’ve probably left it too late anyway,’ and I’ll just wait and see what happens next.* (Woman, 53, positive, not stopped smoking)

### Emotional responses to interpretations of screening test results

Emotional responses to the interpretations described above were central to participants’ motivations about smoking. These responses included fear, shock, upset, worry, anxiety and guilt:

P7: *You don't think there is emotions and fears and anxieties that come up, you think oh it's a study... you're facing something that could be possibly detrimental to you, it can be worrying. For me I think it really reinforces trying to stop smoking.* (Woman, 53, positive, stopped smoking)

P12: *Shocked … shocked but in a round about way … I remember when I got the letter I was crying. I thought ‘oh my God’ … but then when you go back to the place they kinda make you feel better, like saying that it’s nodules and stuff like that.* (Woman, 53, positive, not stopped smoking)

P19: *I felt a bit upset, yeh. Not dreadfully because the nurse I’d spoken to at the hospital said ‘look, it’s not cut and dried, you may get this message saying that there’s, you know, positive result but don’t spare’ [despair] type of thing, so I just took it at her word and sorta went along.* (Woman, 74, positive, not stopped smoking)

P22: *I think I feel worse about being a smoker than I did previously, I’ve always buried my head in the sand about it ... and it kinda makes it more of a reality and it actually makes you feel worse about smoking. Probably more guilty about it actually.* (Woman, 59, positive, not stopped smoking)

Responses to a negative result included relief, reassurance, and indifference:

P10: *Thank goodness ... it was just relief because it come back clear.* (Woman, 54, negative, not stopped smoking)

Some participants experienced a desire to change their smoking behaviour following screening and some wanted to continue smoking. Importantly, there were individual differences in the way in which emotional responses impacted motivations about smoking, with no clear pattern according to test results or interpretations of their meaning. Some were motivated to stop smoking by a positive test result and felt they would have continued smoking if the result had been negative. In others the opposite responses were observed – they were motivated to stop smoking by a negative result but would have continued smoking if it had been positive.

### Interactions between overarching themes and motivations about smoking

We present three examples that demonstrate the link between participants’ interpretations of test results, their emotional responses and their motivations about smoking. The participant in the first example explained how her emotional response to the test result inhibited her ability to understand the risk information provided to her and made her too scared to telephone the study centre to ask questions. She described a vicious circle whereby this emotional response and uncertainty led to her smoke more heavily:

P12: *Have I got it [cancer], or am I going to get it? If I stop smoking will that change, or will I still get it anyway, because of this gene? So there’s a lot of questions, you know. And when you go there for that appointment after it all, you cannae [can not] really take it in, you know you’re sort of sitting talking and you think ‘I must remember that, I must remember that, I must remember that,’ … and I did get a letter I couldnae [could not] even tell you where that is.*

*[...] I’m not quite sure if I’m gonna get cancer or have I got cancer, but I could phone and ask but I’m kind of scared to cause I don’t want to know what they’re gonna say.*

I (interviewer): *So has that uncertainty affected your thinking about smoking at all?*

P12: *Honest to God every time I pick up a cigarette it comes into my mind. It doesn’t matter what I’m doing, every cigarette I light I think about it and I think ‘I’m gonna stop I’m gonna stop I’m gonna stop’ … but I can’t and it’s like a vicious circle where … because you cannae [can not] stop thinking about it you’re smoking more, you know what mean?* (Woman, 53, positive, not stopped smoking)

In the second example (below) the participant’s understanding of his negative test result is that it means he does not have lung cancer but could still develop the disease in the future. He experienced relief, elation and felt lucky. He said this did not change his thoughts about smoking:

I: *Can you remember any time [during the study] where your thoughts or feelings about smoking changed at all?*

P9: *No, I just knew it wasn’t doing me any good, put it that way. It was doing me harm. … But I was relieved to learn that I never had lung cancer but it doesn’t mean to say it wouldn’t recur [occur in the future].*

I: *Could you tell me a bit more about the relieved feeling that you had when you found out that your test was negative? Tell me what that was like when you got the result?*

P9: *Obviously a bit elated, you know, but and lucky. … That’s about it.*

I: *Why did you feel lucky?*

P9: *Well that it had missed me out.* (Man, 67, negative, not stopped smoking)

The participant below experienced a ‘fright’ from a positive test, plus a further fright from a nodule detected on the subsequent CT scan, described by her as the reason she stopped smoking. Having been a smoker for 40 years, her success at stopping surprised her:

P1: *I never thought I would give it up […] so that's really good.*

I: *Why did you say you never thought you'd give up?*

P1: *I don't know I just never thought I would ever stop smoking cos I've tried and tried at different times. It just shows you how a fright like that can really make you stop. And I'm really glad that I went for that [screening]. [...] I'm really glad I done it now. Cos that's what's made me stop smoking. Cos I've got something that's here [in the lung]. I don't even know what it is. The consultant I've seen said I've got something here but it's so many centimetres and they were waiting to see if it grew any more. I had to go back for another CT scan. That's gave me a real fright so that is the reason why I did stop.* [...]

I: *When you found out that your screening test result was positive, can you tell me how you felt at that point?*

P1: *I really got a fright and I didnae [did not] know am I gonna have lung cancer or is it-- you know-- I didnae feel good at all. So I was dying to go back for the CT scan to get that result, to get it all over and done with. So I was quite down at that time you know. Well [research nurse] had said to me that the blood test was positive, wasn't it? That right? So that even gave me a fright at that. She said it doesn't mean you've got any lung cancer or that, but you're in the positive area ... but no, I got a fright at that time as well.*

I: *Did you have any thoughts about smoking at that time?*

P1: *Yeh. That gave me another trigger to stop, you know what I mean? I did want to stop then.*

I: *So at which point did you kind of make that decision that you were going to try and stop?*

P1: *Well after I got the result of the CT scan, that was when I decided that I was definitely going to do it. So that was a few weeks after I got the result that I actually stopped, so, I was really really shocked that there was something there. And I've not asked the doctor, I'm going to phone up and ask exactly what this is. I need to phone and ask does he think it is cancer that's there, do you know what I mean? He says it's right here at the front of my lung, but it is only tiny, he said they had to search the scans to actually find it, so it is tiny, six whatever, I don't know if it's centimetres or--* (Woman, 54, positive, stopped smoking)

These examples show that individuals’ responses were different but that their interpretation of their test result and emotional response were key recurring themes in their motivations about smoking.

The two overarching themes presented above describe the key aspects of how screening influenced motivations about smoking. Further themes presented below elaborate on aspects of the ‘teachable moment’ represented by screening and important social and contextual factors influencing motivations about smoking.

### ‘Teachable moment’

#### Theme: A wake-up call

Screening served as a health scare and a wake-up call, prompting thoughts about the threat of lung cancer. This was described in terms of an objective confirmation of the known risks of smoking:

P22: *Most smokers are very sensible people, you know the risks that you’re taking but it’s a very concrete thing isn’t it when you get a test result like that, well it’s concrete in some ways, the reality of what you’re doing to yourself.* (Woman, 59, positive, not stopped smoking)

Another participant expressed surprise that a positive result, interpreted as meaning her life was at risk, hadn’t caused her to decide to stop smoking:

P2: *Even after I found out that I did have a positive result and both lungs have got nodules ... I’m still smoking! I mean I never ever ever thought that I would do that. I thought any time when it comes, I’ll have to make a decision and I’ll make it and that’ll be it, you know, when my life’s at risk right away I’ll make it and that’ll be it and I haven’t done that.* (Woman, 60, positive, not stopped smoking)

A participant expressed a desire for the receipt of a forceful message from a doctor for an even bigger wake-up call and additional motivation to stop smoking:

P12: *All the doctors need to say is, ‘Listen, you. You’ve came back with a positive result. If you dinnae [do not] stop smoking today, right now, you’re gonna die.’* (Woman, 53, positive, not stopped smoking)

#### Theme: Now is the time to stop smoking

Screening created a sense that ‘now’ was the right time to attempt to stop smoking, although this was not always acted upon:

P20: *I says now I've got the chance to stop it but I didnae [did not], know what I mean?* (Man, 56, negative, not stopped smoking)

Another participant described being even more determined to stop after being told that the chest x-ray was clear:

P5: *You stop now before you make it worse. You’re okay at the moment, you’re not a hundred per cent but it could be a lot worse. Now’s the time to stop.* (Man, 62, negative, stopped smoking)

### Social and environmental context

#### Theme: Family influences

Following screening, family members were influential in guiding individuals’ motivations about smoking:

P27: *The reaction of my family [to the screening result], in particular my husband, he was so upset, he was even worse than me to be quite honest with you. He still never turned round and went like that ‘well you’re gonna have to stop smoking’ or anything like that, I done that myself.* (Woman, 63, positive, stopped smoking)

One participant explained how pressure from his wife, combined with the test result, convinced him to try to stop smoking:

P29: *She’s been on at me for years to stop smoking and I think a combination of her plus the study plus the fact that I was lucky enough that it was clear that I may be chancing my luck if I keep on going.* (Man, 70, negative, stopped smoking)

The offer of information about local smoking cessation services by research nurses was not mentioned by participants during interviews or identified as a theme influencing motivations about smoking.

#### Contextual factors

There were important non-screening factors influencing smoking motivations in this sample. These included age and life stage factors such as becoming a grandparent, respiratory symptoms, and financial factors. These themes are described in Table 3 with example quotes.

**Table 3:** Themes not specific to lung cancer screening and example quotes

Theme	Example quotes
Age and life stage	P4: *As I’ve got older I suppose there is an effect in the sense that it’s all very well saying ‘I’ll carry on smoking’ and then you die, I’ve been more lately thinking, well yeh but it might be a long painful death.* (Man, 58, negative, not stopped smoking) P5: *My own mortality, reaching 62 and thinking ‘oh you’re nearer the end than you are the beginning now, you’d better watch what you’re doing,’ that sort of thing. When I go I want to die in my own home, reasonably fit, and I thought if I keep smoking that might not happen. When you start reflecting and you get near the age you were when they [participant’s parents] died and you think ‘maybe it’s time you stopped’.* (Man, 62, negative, stopped smoking) P6: *My first grandchild was expected. I just did not want to be a smoking granny.* (Woman, 71, positive, stopped smoking)
Respiratory symptoms	Interviewer: *Can you remember what made you decide to try and stop back in January?* P26: *Because truthfully it’s my health, it’s my health. Cos like I don’t feel ill, it was more when I lay in my bed at night I could hear myself wheezing and I said ‘need to give up these cigarettes I’m gonna end up really ill,’ and you know? And I think that was one of the reasons.* (Woman, 57, negative, not stopped smoking)
Money	P4: *My motivation in trying to stop smoking was really financial. It’s now something like seven pound fifty a packet, incredible price you know, so that that was the real motivator I have to say.* (Man, 58, negative, not stopped smoking) P18: *The main reason I would like to stop is the money aspect, cos it is very very expensive and I mean it probably sounds really daft, I mean I should be thinking more about my health but I think more about the money aspect of it because I do enjoy a cigarette.* (Woman, 58, negative, not stopped smoking)
Pre-screening decisions to stop smoking	P5: *And I thought this [ECLS study] is just another way of trying to stop so I’m going to go for it and see what happens. I didn’t know what it was all about then, obviously.* Interviewer: *So you thought it could help you to try and stop smoking?* P5: *Yep I needed motivation to stop. And you can do with any motivation you can get.* (Man, 62, negative, stopped smoking)

### Coinciding factors

Some said the screening had motivated them to stop smoking in combination with coinciding non-screening factors:

P7: *Everything sort of fitted in at the right time for me because ... before I had a wee scare and I kept thinking ‘oh I want to stop smoking’, different things had happened [family bereavement] and so it all seemed to—* (Woman, 53, positive, stopped smoking)

P6: *I felt that the stars were aligned if you like, I had the Champix [medication to treat nicotine addiction], I had [smoking cessation counsellor], I had my granddaughter all as the sort of incentive and I thought I might never be so lucky again as to get that that combination of things all at once. It was a combination of things and I think getting this positive result in a way was sort of marginal, it may have been like a sort of final thing.* (Woman, 71, positive, stopped smoking)

Lung cancer screening was a novel experience that, in combination with other factors, provided an opportunity that they felt might not be available again. Screening was described as having come at the right time and having ‘brought it all in’ and ‘brought it all to a head’ in relation to other motivating factors.

Of those who did not want to stop smoking there was often a lack of detail in their accounts of their motivation to continue smoking as it was not something they had given much thought. Three themes we extracted from these participants’ data are described in Table 4 with example quotes.

**Table 4:** Themes in those who did not want to stop smoking

Reassurance from study schedule CT scans	P14: *If they see any changes within the CAT scan it’s gonna be caught at an extremely early stage. If there’s any changes well, I’ll just cross that bridge when I come to it.* (Man, 64, positive, not stopped smoking)
Too late to stop now	P19: *I suppose at my age lack of motive. I mean I’ve known quite a number of people younger than me have died. I don’t expect to live that much longer and I’d rather live it pleasantly.* (Woman, 74, positive, not stopped smoking) P14: *I think I’ve had a good life and I’ve been here long enough and I think now if it was going to be something serious well, what would I get out of it? Another 3 or 4 years, you know. I’m not unduly perturbed about it, the prospect. Sad to say.* (Man, 64, positive, not stopped smoking)
Avoidance of thoughts about smoking	P26: *I blank it out my mind, smoking. I blank lung cancer out my mind.* (Woman, 57, negative, not stopped smoking) P22: *I think it [smoking] probably is always in my mind but I am a bit of a bury my head in the sand kinda person about it.* (Woman, 59, positive, not stopped smoking)

**Fig. 2:**
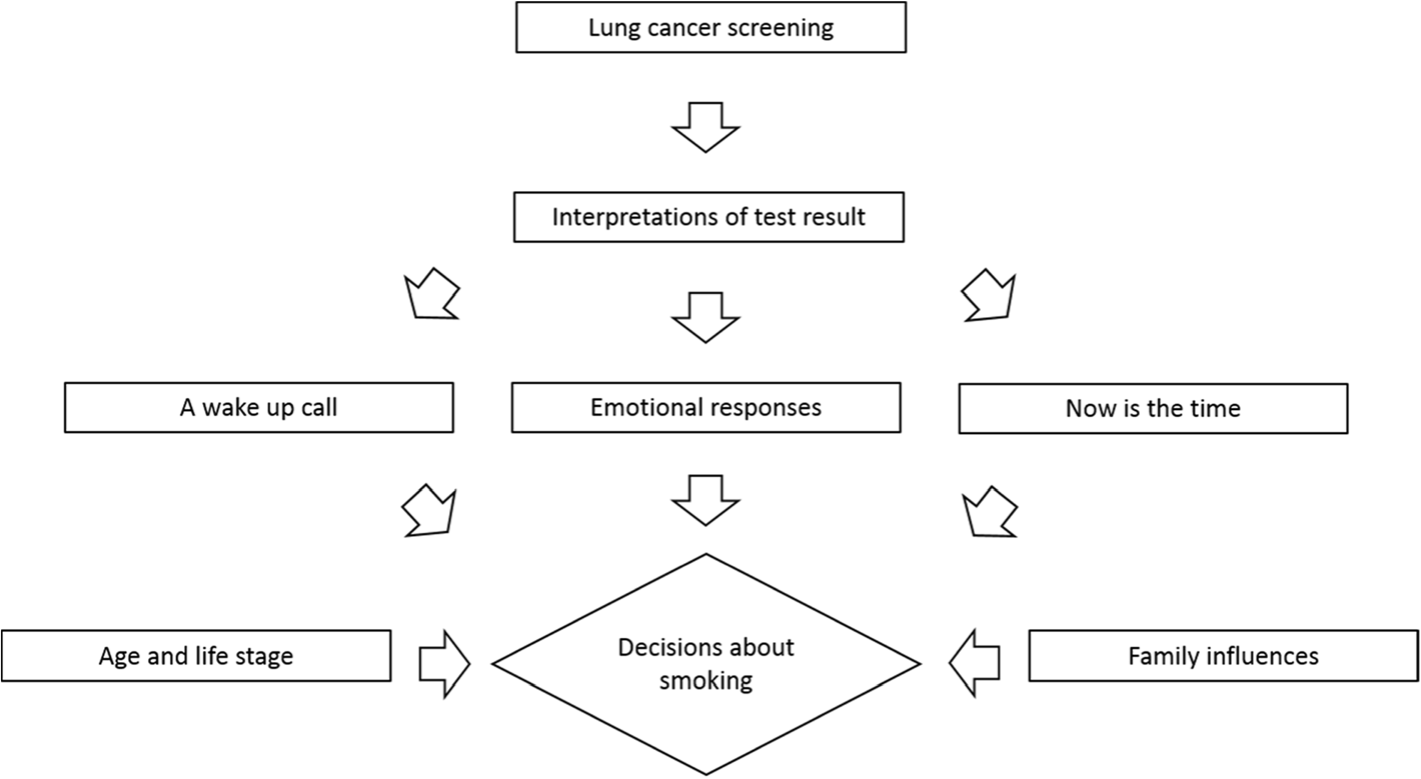
Diagram of themes

Cues to change were experienced at different stages of the screening process, not always immediately following a test result. Some people already had a desire to stop or cut down smoking before screening and their screening experiences either reinforced or did not change these desires (Table 3). Participation in lung cancer screening led to other smoking behaviour changes, for example several participants had tried to cut down but had not attempted to stop. One participant had been prompted by their screening experiences to begin using filters in their roll-up cigarettes but had not had any thoughts about stopping smoking. A diagram of themes is shown in Fig. 2.

## Discussion

This study is the first to our knowledge to explore in-depth how lung cancer screening influences motivations to quit smoking and the first to purposively recruit individuals that have stopped smoking after lung cancer screening.

### Principal findings

Motivations about smoking were closely related to emotional responses to test results, which in turn were linked to how individuals interpreted these test results, often inaccurately. Because people had different levels of understanding about what the test result meant, different emotional responses to those understandings and different pre-existing motivations to change their smoking behaviour, their motivations about smoking were individualised and difficult to predict. We found positive and negative results were both experienced as a reason to stop smoking or a reason to continue smoking. Screening was a ‘wake-up call’ to the risks of smoking and created a sense that ‘now’ was the time to stop smoking. Family members, age-related factors and the existence of multiple coinciding non-screening factors were also influential. The teachable moment was experienced at different stages of the screening process and some had participated in screening in order to try to stop smoking. Those who did not want to stop smoking experienced reassurance from study scans, felt that it was too late to stop, or avoided thinking about stopping.

### Strengths and limitations

Qualitative methods allowed an in-depth and nuanced exploration of the patient perspective of the topic. There was a good response rate to study invitations. Rigour was demonstrated by the inclusion of those who had received positive and negative test results and those who had and had not tried to stop smoking, along with the use of an inductive and deductive approach to analysis. Reliability was ensured by digital recording and verbatim transcription of interviews, the use of software to allow systematic coding, and discussion between two researchers during the sorting of codes into structures to generate overarching themes. We demonstrated sensitivity to context by positioning the study within the wider ECLS study and through the use of neutral, non-judgmental language in approaching the topic of smoking in the invitation materials and interviews [32].

Some limitations should also be considered. Participants were likely to be more motivated to stop smoking than the wider smoking population because they had agreed to take part in a screening study, a nested questionnaire study and a further qualitative study. Having a blood sample taken for cancer screening could have been an unusual experience for them and the results may be less generalisable to screening programmes involving just CT without a preceding blood screen. During recruitment to the ECLS study, participants were provided with information about concepts such as randomisation and allocation to the control group, which can cause confusion [28] and could have inhibited understanding of other information such as the meaning of test results. We did not have data on patient self-reported health-literacy or numeracy to explore whether this influenced interpretations of test results. Finally, most participants asked if the interviewer was a smoker and may have adjusted their explanations about smoking after learning that this was not the case. The researcher did, however, take a neutral standpoint on any issues arising and as a visitor to the study regions could be distanced from the local ECLS study clinical processes and engage simply as an interested outside observer of experiences.

### Comparison with other data

Individualistic responses to screening test results can help explain why, except for those receiving abnormal results in some studies, consistent patterns in the impact of lung cancer screening on smoking have not been observed to date. Misinterpretations of the degree of lung cancer risk implied by positive or negative lung cancer screening results is a novel finding that highlights complexity in the behavioural impact of lung cancer screening. It suggests that any change in motivation for smoking cessation created by lung cancer screening may sometimes be based on a suboptimal understanding of information provided to screening participants. This provides support for concerns raised about the effects on patients of dichotomising cancer screening test results into ‘positive’ and ‘negative’ [33].

The finding that lung cancer screening test results are experienced emotionally, and that this can influence health behaviour, has been reported in previous work. A qualitative study of CT lung-screened smokers reported emotional arousal as one of three key pathways by which screening may influence motivation around smoking cessation [26]. Abnormal lung screening results have been shown to have a short-term adverse impact on emotional outcomes [34–37] and to promote smoking cessation [38]. Uncertainty management theory can provide a framework around which to understand the relationship between emotional and behavioural responses to screening. Screening test results can change an individuals’ level of uncertainty about their health, their appraisal of this uncertainty can elicit a positive or negative emotional response, which can influence smoking behaviour [39]. In smokers undergoing lung cancer screening these emotional responses might create active or passive dissonance with emotional responses to smoking behaviours. In this way, smokers’ motivations and decisions about smoking are a response to screening resulting from their appraisal of uncertainty and the emotions that result. In our participants the uncertainty they experienced relates to their understanding of screening test results. Furthermore, their emotional response to uncertainty appraisal can lead to information seeking or avoidance and in the case of information seeking behaviour, this could improve understanding of screening test results.

The themes ‘wake-up call’ and ‘now is the time to stop smoking’ support the idea that lung cancer screening can be a teachable moment for smoking cessation. This contrasts with evidence from the National Lung Screening Trial, which reported screening was not a cue to action and high risk perceptions were not related to quitting [25]. This may be due to our study adopting a more in-depth and loosely structured approach to data collection, allowing the wider context of smoking motivations to be explored. For example, we found when lung cancer screening played a role in motivations to stop smoking there were often other important non-screening factors at play.

Some had taken part in screening in order to try and stop smoking. This is consistent with a previous qualitative finding that motivation to stop smoking was one of three perceived benefits of lung cancer screening in smokers [40]. We also found some evidence that reassurance from CT scans could reduce motivation to quit, and that some people cut down their smoking or made other changes, both findings that have been reported previously [25, 26].

### Implications for research, policy and practice

The polarised way in which screening test results were sometimes interpreted in our study (very high/very low risk) was a factor in motivations about smoking. To aid understanding, ECLS study screening test results were communicated as ‘positive’ or ‘negative’ along with probabilities using simple frequencies. However, there were still deficits in understanding, highlighting a need for further progress in this area to enable better understanding of lung cancer screening tests and test results by participants. Research to describe the complexities of experiences of uncertainty in lung cancer screening patients who smoke can help to develop communication processes that facilitate desired behavioural responses in the management of that uncertainty. This can include information seeking and smoking quit attempts.

Smokers may experience different emotional responses and motivations about smoking if they understand their risk in a different way. It is therefore important that quantitative studies of the impact of lung cancer screening on smoking account for levels of perceived risk. Any assessment of the overall benefits and harms of a lung screening programme should consider how well test results are understood and how individuals might react emotionally and behaviourally to those results. Furthermore, when considering the emotional harms of screening it should be acknowledged that short-term emotional harms could promote longer term benefits such as smoking cessation.

The findings suggest that smoking cessation advice in lung cancer screening should be tailored according to individual interpretations of, and emotional responses to test results. A randomised trial of male smokers found computer-tailored advice did not result in significantly different abstinence rates than standardised advice following lung cancer screening [18]. Importantly, the advice was tailored to smoking attitudes and behaviour but not to understanding of and emotional response to screening test results. A telephone counselling intervention which aimed to use lung cancer screening test results to increase risk perceptions was effective at promoting cessation in a pilot randomised trial [20]. The stated aim of this strategy was to capitalise on the teachable moment of an abnormal result and to counteract the potential for reduced motivation to quit after a result showing no nodules or abnormalities. A responsive approach such as this, rather than a computer-tailored method, has the flexibility to adapt the advice depending on the attitudes and intentions of the individual, which our study showed can be unpredictable and individualistic. Such interventions should also be flexible in the timing of delivery and should be offered after the test result is delivered so that interpretation of the result can be explored and emotional responses can be further pursued. Family members could also be involved and non-screening factors addressed. The finding that those who did not want to stop smoking sometimes felt it was too late to do so requires further exploration to ensure this perception can be addressed. Further research is needed to explore what type of cessation support lung cancer screening participants who smoke would find most acceptable and useful.

## Conclusions

Our study demonstrates individualised and complex motivations about smoking among lung cancer screening participants and the ways in which lung cancer screening can create a ‘teachable moment’ in motivations about smoking. Emotional and behavioural responses to test results, which can be misinterpreted, varied between individuals. Lung cancer screening presents an opportunity to engage high risk smokers in cessation attempts but cessation support may need to be tailored to an individual’s emotional response to their understanding of their test result and take account of the range of factors we have identified to be most effective.

## Additional file

Additional file 1: Interview schedule. Question guide for semi-structured interviews. (DOCX 14 kb)

## Abbreviations

CT: Low-dose computed tomography
ECLS: Early Cancer Detection Test - Lung Cancer Scotland

## Footnotes

1 unique indentifier

2 gender

3 age (years)

4 EarlyCDT-Lung test result

5 post-screening smoking cessation as described by participants in interviews (stopped smoking/not stopped smoking)
