# Supplementary material for: Determinants of motivation to quit in smokers screened for the early detection of lung cancer: a qualitative study
Source: BMC Public Health. 2018 Nov 20;18:1276. doi: 10.1186/s12889-018-6211-1 (PMC6245764; doi:10.1186/s12889-018-6211-1)
Supplement: Supplementary file 1 — Interview schedule. Question guide for semi-structured interviews. (DOCX 14 kb) [file 12889_2018_6211_MOESM1_ESM.docx]

**Determinants of motivation to quit in smokers screened for the early detection of lung cancer: A qualitative study**

**Additional file 1**

**Question guide for semi-structured interviews**

Aim: To identify thoughts, feelings and experiences about being screened for lung cancer

1. How was your experience of taking part in the ECLS study?
2. How did you find out about the study?
3. Were you aware why you had been invited to take part?
4. What were your reasons for wanting to take part?

Aim: To establish smoking history and previous cessation attempts

1. Can you tell me about your smoking history?
   1. When did you start smoking?
   2. Have you ever stopped/tried to stop/cut down smoking?
      1. How did that go?
      2. How long did you stop smoking for?
      3. Did you use any particular strategy?
      4. How easy or difficult did you find it?
      5. How did you feel about that?
      6. How long ago was that?

Aim: To establish decisions made regarding smoking since finding out about the ECLS study, the success of those decisions and explore reasons for those decisions and perceived barriers and facilitators to cessation.

1. *(for participants who tried to stop smoking since the study)* Can you tell me about your decision to try/stop smoking?
   1. Which method(s) did you use to try to stop smoking?
   2. How easy or difficult was it for you to try to stop smoking?
   3. What do you feel helped you to try to stop smoking? (explore in depth)
   4. Which things did you feel did not help you to try to stop smoking? (explore in depth)
   5. Was there any time during the study that your thoughts or feelings about smoking changed?
      1. When was that?
      2. How did your thoughts or feelings change?
      3. Why do you think your thoughts or feelings changed/did not change?
2. *(for participants who did not try to stop smoking)* Did your thoughts and feelings about smoking change at all during your participation in the ECLS study? If so, how?
3. *(for participants who did not try to stop smoking)* We know that some people find that having a lung cancer blood test makes them want to stop smoking but other people find that it doesn’t. It is important for us to understand why this is. Based on your experience of having a lung cancer blood test, why do you think this is?
4. If your screening test result had been [positive/negative] (explain briefly what that would mean) would you have felt any differently about smoking?
   1. In what way?

Aim: To explore thoughts and feelings about smoking cessation advice for lung cancer screening patients.

1. Imagine you are having a lung cancer blood test for the first time, but this time everybody who has the test is given special advice and support about stopping smoking. How would this make you feel about smoking?
   1. Would it make you think or feel differently about having the lung cancer blood test if you knew this was going to happen? If so, how?
   2. Would it make you think or feel differently about stopping smoking? If so, how?
   3. Would it change how confident you felt about being able to stop smoking? If so, how?
   4. Would it change your plans to stop or carry on smoking? If so, how?
   5. Would it make you more likely or less likely to have a repeat lung cancer blood test in the future e.g. five years’ time? And why?
2. What type of special advice & support would you find most helpful, if it was offered to you during a visit for a lung cancer blood test?
3. Is there anything else you would like to tell me about the things we have talked about?

*If participant is not forthcoming with thoughts or feelings about smoking, explore their responses to the study questionnaire smoking items e.g.* You said you were fairly uncertain that if you tried, you could give up smoking for good? Why did you select that answer?
